# The moderator effect of the family business protocol on the financial performance of born globals firms: A trajectories analysis

**DOI:** 10.1371/journal.pone.0332771

**Published:** 2026-11-04

**Authors:** Diana Escandon-Barbosa, Jairo Salas-Paramo

**Affiliations:** 1 Facultad d’ economía I’ empresa, Universitat Autònoma de Barcelona (UAB), Barcelona, Spain; 2 Faculty of Economic Sciences and Administration, Pontificia Universidad Javeriana-Cali, Santiago de Cali, Colombia; IU Internationale Hochschule GmbH, GERMANY

## Abstract

Born global firms (BGFs) face unique challenges in achieving financial stability due to their rapid internationalization and resource constraints. While family-owned BGFs may benefit from long-term orientation and family firm protocol structures, the impact of a family business protocol (FBP) on their firm’s performance remains underexplored. This study investigates the triple moderating effect of FBP on the firm performance trajectories of BGFs, considering how market uncertainty, formal family firm protocol mechanisms shape strategic decision making (Absorptive capacity) and firm performance over time. With a longitudinal approach, we analyze a dataset of family owned BGFs across multiple industries, applying trajectory analysis to assess variations in performance under different family firm protocol structures. Our findings suggest that the presence of an FBP improve financial resilience through reducing decision making conflicts and at the same time increasing strategic consistency, particularly in firms operating in volatile environment in international markets. However, excessive formalization may also impose rigidity, potentially limiting adaptability in highly dynamic environments. In this way this study contributes to the literature on international entrepreneurship and family business by offering new insights into the role of formal family firm protocols in shaping the financial trajectories of BGFs. The findings offer valuable implications for family business owners, policymakers, and scholars seeking to optimize family firm governance structures for sustained financial growth in globally oriented firms.

## Introduction

According to the European Commission [1], family firms account for 60% of the total number of companies in Europe. Their size and composition are very diverse, ranging from sole proprietorships to large enterprises. According to the above, their role in the European economy is significant, as they create conditions not only to drive market growth and development but also to provide an infrastructure that supports their activities. Family firms are defined based on the criterion of legal decision-making rights held by the founder and family members, who directly or indirectly hold control and are involved in the family firm protocol, and where at least 25% of the rights are held by the family [1].

An important element to highlight is that family firms in the current scenario tend to face various challenges, including the need for businesses to prepare for transfers, financial access, taxes, and the balance of family firm protocol between family and business, attracting and retaining skilled workers, and developing entrepreneurial education for family business management. According to the [1], it is important to highlight that these types of firms have support mechanisms at their disposal that allow them to access structural investment funds that seek to promote an environment of information dissemination in family businesses to encourage best practices among the countries that are part of the European Union. It also calls for the adoption of tax systems and corporate laws that promote entrepreneurial activity through business education and support.

Related to the research literature on family firms and firm performance, this type of research can be grouped into several topics that highlight different dimensions of family firm protocols, for example, innovation and strategic behavior. One of these categories is family firm protocol and its impact on firm performance [2], Corporate family firm protocol frameworks in family firms [3–5], heterogeneity of family firm protocol mechanisms [5], intersection of family involvement and innovation [6], and formalized family firm protocol through family constitutions [7].

Literature exploring absorptive capacity in family firms can be organized into several thematic categories that reflect the complexity and evolution of this research domain. One of the most important categories focuses on the relationship between absorptive capacity and innovation, as seen in the work of Hernández-Perlines et al. [8] and Hernández-Perlines et al. [9], who analyze how entrepreneurial orientation and dynamic capabilities enhance innovation and business performance in family businesses.

On the other hand, an important stream is represented by systematic reviews and conceptual frameworks, such as the study by Pütz and Werner [10], which synthesizes the existing literature and identifies theoretical gaps in understanding absorptive capacity within family firms. A third category addresses absorptive capacity as a driver of strategic and sustainable outcomes, with Tufan and Mert [11] examining its sequential effect on agility and sustainable performance, and Belkhodja [12] exploring its role in managing knowledge as both a challenge and an opportunity. The micro-level perspective on managerial roles is also emerging, with Eniola [13] emphasizing the function of small family firm entrepreneurs as knowledge gatekeepers. Conversely, a theoretical category combines family, entrepreneurial orientation, and absorptive capacity, as illustrated by Santos et al. [14], highlighting how these constructs interrelate in shaping knowledge systematization in family firms. These categories reveal a maturing field that integrates behavioral, strategic, and operational perspectives on how family businesses absorb and leverage external knowledge.

Among the gaps identified, the first relates to the weakness in analyzing the role of family protocols and how they serve as governance mechanisms in dynamic environments [2,5]. This approach highlights the need for empirical analysis of family protocols, including agreements, roles, and methods for conflict resolution and succession in the context of market uncertainty. The second gap is related to the interaction required to explore absorptive capacity and family firm protocol structures. Although research on absorptive capacity and its relationship with innovation and performance is varied, few studies have focused on the effectiveness of forming family firm protocol mechanisms for family businesses that impact through protocols and decision-making systems. Considering the potential moderation of family protocols allows us to glimpse a field that needs to be explored, especially in environments characterized by high uncertainty [8,10].

The research examining market uncertainty and family firms can be grouped into four main thematic categories, reflecting diverse strategic, behavioral, and institutional dynamics. First, a central category examines the role of family firm protocol, power, and executive influence in uncertain environments, with studies such as Deng et al [15] and Xu et al [16] analyzing how CEO power and sibling co-management impact financial decisions, including mergers, acquisitions, and the cost of capital, in uncertain markets.

A second category emphasizes the intersection of social capital, political dynamics, and cultural conformity, where scholars like Qi et al. [17] and Yang et al. [18] highlight how CEO social ties and political alignment can stimulate the effects of uncertainty in family firms. A third categorical area focuses on socioemotional wealth and stewardship behavior in navigating turbulent environments, as examined in Åberg et al. [19] and Sharma & Sharma [20], which analyze how family values and long-term orientation influence responses to uncertainty.

Finally, a growing stream addresses CSR and strategic adaptation, illustrated by Anwar & Idris [21], whose findings reveal how CSR can improve investment efficiency during economic policy uncertainty, particularly distinguishing between family and non-family firms. These categories reveal how family firms adopt multifaceted strategies balancing tradition, innovation, and relational capital to maintain resilience in volatile markets.

Thus, the purpose of this research is to analyze the effects of the double moderation effect of the family firm protocol in highly uncertain markets on the relationship between absorptive capacity and firm performance in family firms in the born global sector. Based on the above, the following research question is suggested: How does family firm protocol in highly uncertain markets influence absorptive capacity (assimilation, transformation, and exploitation) in firm performance? To address the purpose and research question, the paper is structured as follows. The first part highlights the significance of family firms and the advancement of their field of knowledge, drawing on the most recent research. The second part presents the theoretical framework that allows for arguing the relationships within the model. The next component presents the methodology used in the study, explaining the data analysis technique and how the sampling was conducted. The following section presents the main findings of the research, followed by the conclusions and the study’s key theoretical and empirical contributions.

### Theoretical framework

Stewardship Theory remains an essential framework in understanding family firm protocol in family businesses, particularly because it shifts focus from purely self-interested, transactional relationships to those driven by intrinsic motivation, trust, and long-term commitment. In the case of agency theory, which assumes individuals act opportunistically to maximize personal utility, stewardship theory posits that managers, especially in family firms, often act as stewards who prioritize organizational and familial goals over short term selfgain.

This theoretical lens is particularly relevant in family businesses, where relational continuity, socioemotional wealth, and generational legacy frequently guide decision-making. Although critics argue that stewardship theory may offer an overly idealized view of human behavior, assuming individuals are consistently altruistic, it nonetheless highlights the limitations of family firm protocol systems grounded solely in control and incentive mechanisms. Through different research finding scholars acknowledge that individuals embody both agentic and steward-like tendencies influenced by organizational culture, leadership values, and situational factors.

In this sense, stewardship theory’s most significant contribution lies in emphasizing the importance of trust, shared purpose, and relational protocols within family firms. When complemented with mechanisms for transparency and accountability, stewardship based family firm protocol can enhance alignment between ownership and management, particularly in complex contexts such as internationalizing family firms or born globals. Therefore, while imperfect, stewardship theory offers critical insights into non-economic drivers of performance and sustainability.

On the other hand, stewardship theory specifically assumes that opportunistic behavior is driven by the intention of pursuing self-interest [22]. Despite this, it is argued that if both parties are considered utility-maximizing actors, it is a strong argument to establish that not all actors will necessarily engage in opportunistic behavior [23]. An important point to note is that, considering the REMM model, an individual will constantly seek to fulfill their interests in terms of being recursive, evaluative, and maximizing their action model. This implies that people will have unlimited preferences and desires and will engage in trade-offs, pursuing maximum satisfaction by responding to incentives such as money [24].

From the perspective of agency theory, this perspective is related because its basis is wholly based on human behavior, which entails a direct dependence on the organizational family firm protocol of firms, especially in family businesses [25]. An important point to highlight is that stewardship theory posits that most people can adjust their behavior according to self-interest and potential opportunistic behavior, which varies depending on the situation and time. However, scholars such as Hernandez [22] note that behavior can also be influenced by the principles of administrative management or firm philosophies, considering risk orientation, time frame, objectives, reward motivations, identity, and power.

Stewardship theory posits that family firm protocol systems are designed to address organizational challenges, where parties involved in decision-making processes between owners and managers create conflicts between different power groups. The focus is on maintaining appropriate relationships at various levels of organizational life [26]. In this regard, the relevance of stewardship theory is based particularly on the challenge of understanding organizations not only as a complete conflict resolution mechanism but also on the possibility of providing managers with insights into opportunistic behavior over a time frame, thus providing an unrealistic and theoretically representative view of how organizational family firm protocol works [25].

Despite the similarities with agency theory, there are also problematic elements that stewardship theory attempts to overcome. One of these incentives and control systems is difficult to detect and correct over time. This is because agents can have a negative influence on behavior. The above suggests that although individuals have intrinsic motives to direct their actions, it does not mean that those actions will be based on organizational objectives due to the possible absence of a principal and the lack of direction and feedback [25]. In conclusion, stewardship theory, like agency theory, offers a valuable perspective on reality by capturing the influence of personal interests, motivations, and other factors on the motivations of family firms. Likewise, this model enables the capture of the varied motivations of managers and employees, as well as their context, in both family and non-family businesses, highlighting the importance of factors related to family protocols, such as absorptive capacity, and their impact on firm performance.

### Family firm protocol, Absorptive capacity, and firm performance

One of the more important characteristics in family business management is the volatile environment and intensive knowledge related to the business dynamics. Understanding the mechanisms through which firms absorb and apply external knowledge is crucial for advancing both theory and practice in strategic management.

Among these mechanisms, absorptive capacity has emerged as an important construct in explaining innovation and superior performance outcomes [27,28]. However, despite its prominence in organizational theory, absorptive capacity cannot be fully understood in isolation.

To strengthen its explanatory power, scholars in the area increasingly call for its integration with contextual variables that shape how knowledge is internalized and leveraged in firms, particularly family firms, where non economic goals and relational dynamics have a significant influence on their management decisions [29,30].

Theoretically, one of the most promising frameworks for exploring this contextual influence in family firms is the stewardship theory, which emphasizes trust-based, participatory, and long-term oriented family firm protocols. In contrast to agency theories that focus on opportunism and control, stewardship theory offers a nuanced perspective on organizational actors, especially in family owned firms, where alignment between ownership and management promotes conditions for effective knowledge absorption [31,32].

Family firm protocol structures rooted in stewardship values serve as either amplifiers or inhibitors of absorptive capacity, depending on how they condition organizational behaviors and resource flows. Thus, integrating family firm protocol as a moderating variable is not only theoretically relevant but also necessary to understand the boundary conditions of absorptive capacity’s influence on performance.

An important aspect to highlight is that family firms are defined based on the possibility of business continuity to the next generation or the entry of new generations [33]. Within the dynamics of these businesses, obstacles and conflicts between generations can be found, influencing not only business dynamics but also value creation processes. Similarly, problems arise in the adoption of new technologies to take advantage of new market opportunities [34,35]. However, these types of opportunities can be leveraged as long as the structure of family firms is sufficiently developed to adapt to their specific contexts.

It is precisely in this context that measurements such as the FPEC (Family Power, Experience, and Culture) regain importance. The FPEC is considered a multidimensional scale designed to measure the influence of family business structures through three components: power, experience, and culture. This scale has been widely used from a research perspective, allowing approaches to quantitatively assess the impact of family dynamics at the organizational level [36].

In the case of power, it is related to the structure of making decisions between different stakeholders who belong to the family. Power is also related to the capacity of every member of the family to impact the different strategies that are built to define the way of the firm. This is also related to variables that could be important for analyzing conflicts and the different arrangements that need to be made to preserve the health of the company. The second factor is experience, which is related to the knowledge that family members possess and becomes a critical factor for making decisions. This factor also considers the knowledge of the different strategies that have been important in the history of the firm. Finally, culture is considered a set of beliefs that influence the behavior of family members and includes different elements that constitute organizational life.

Field research in family firms emphasizes core aspects of the nature of family firms, such as succession plans and the distribution of power within the firm. Thus, the FPEC establishes dimensions necessary for analyzing strategic decisions and, in particular, firm performance in the context of internationalization, as it relates to born global firms. Similarly, in the field of applying the FPEC, longitudinal analyses are found that allow for the exploration of elements such as power, experience, and culture, and their impact on the effectiveness of family protocols. This constitutes an important line of development related to the application of theory and practice in the context of family businesses, especially the related market dynamics.

In the case of a family firm’s environment, market uncertainty has become particularly relevant in terms of the firm’s management and decision-making. An additional layer of theoretical complexity is introduced that has been underexplored in absorptive capacity research. The strategic behaviors that firms adopt in uncertain environments are mediated by their responses to volatility within the family firm protocol, whether they employ rigid, control-based mechanisms or adaptive, trust-based strategies. Studies suggest that family firm protocols under uncertainty can either enhance or constrain absorptive processes, depending on their flexibility and responsiveness [37,38]. Therefore, positioning market uncertainty as a second-order moderator enables a more dynamic and realistic understanding of how knowledge capabilities interact with both internal family firm protocols and external volatility.

In this way, the present research proposes a multi-layered model that integrates stewardship-based family firm protocol, the absorptive capacity, and market uncertainty to explain variation in firm performance. The proposed framework contributes to theoretical advancement in three ways: (1) by contextualizing absorptive capacity within the relational and cultural fabric of family businesses, (2) by reconceptualizing family firm protocol not as a static control mechanism but as a dynamic moderator influenced by stewardship principles, and (3) by introducing market uncertainty as a contingent condition that reshapes how family firm protocol and absorptive routines co evolve.

Research in family firms can benefit significantly from this integrative perspective. By bridging micro-level behavioral assumptions (as in stewardship theory) with macro-environmental challenges (such as uncertainty), scholars can develop more comprehensive, context-sensitive models of organizational performance. This theoretical integration also opens avenues for longitudinal, comparative, and mixed-methods research designs that explore how firms adjust their knowledge strategies and family firm protocol practices in response to changing economic and institutional landscapes.

In the case of firms with high absorptive capacity, especially those that effectively assimilate, transform, and exploit knowledge, they demonstrate superior innovation and financial performance outcomes [27,28,39]. This capability is crucial in enabling firms to navigate complexity and dynamically respond to external changes. In family businesses, the ability to internalize and act on external knowledge is crucial for achieving long-term goals, enhancing innovation capabilities, and sustaining a competitive advantage [29,30]. The strategic role of top management teams in shaping absorptive capacity further enhances a firm’s responsiveness and knowledge utilization [31]. These dynamics underline the centrality of absorptive capacity as a driver of organizational performance, particularly in knowledge-intensive and uncertain environments [40,41]. By the above, the following hypothesis is proposed:

H1. Absorptive capacity (assimilation, transformation, and exploration) has a direct relationship with the firm’s performance.

According to stewardship theory, a family firm protocol grounded in shared purpose, relational trust, and non-opportunistic behavior enhances the productive use of absorptive capacity. When power is appropriately distributed, top managers possess relevant experience, and organizational culture emphasizes long-term orientation, family cohesion, and participative leadership, firms create conditions that encourage knowledge sharing and experimentation. Empirical evidence suggests that well-connected core management teams utilize their network ties to transform knowledge inflows into green innovations [32]. Additionally, digital transformation initiatives, coupled with internationalization, enhance ESG outcomes when family firm protocols channel learning toward sustainability goals [38]. Likewise, firms that combine big-data analytics capabilities with intense absorptive routines achieve superior competitive advantage in dynamic agribusiness markets [42]. By contrast, family firm protocol structures marked by excessive power concentration or calculative, low-trust relationships can impede knowledge flows and invite supplier opportunism, diminishing the benefits of absorptive capacity [43]. Open-innovation studies further indicate that stewardship-oriented family firm protocol helps firms translate external knowledge into higher overseas income while simultaneously boosting environmental and social performance [44]. Collectively, this literature underscores that an effective, trust-based family firm protocol is a critical boundary condition shaping how absorptive capacity translates into firm performance. In this sense, the following hypothesis is proposed:

H2. The family firm protocol (encompassing power, experience, and culture) has a moderating effect on the relationship between absorptive capacity and firm performance.

Market uncertainty introduces external volatility that challenges firms’ strategic decision-making, but its impact depends on how family firm protocol structures channel knowledge inflows. When stewardship-oriented family firm protocols emphasize adaptability, trust, and a long-term vision, family firms can buffer disruptive shocks and more effectively transform dispersed knowledge into a competitive advantage [37]. Depth-of-openness studies show that under high uncertainty, firms with flexible appropriability mechanisms reap stronger performance gains from absorptive capacity than those with rigid, control-heavy regimes [38]. Conversely, agency-driven family firm protocols marked by power concentration, opportunism, or risk aversion often limit knowledge sharing and curtail the benefits of absorptive routines, especially when market signals are ambiguous [45]. Empirical evidence from turbulent emerging-market contexts further suggests that first movers with adaptive alliances outperform their rivals only when family firm protocols support rapid learning and renegotiation [46]. Similarly, firms sourcing external technology under high uncertainty tend to favor flexible, trust-based family firm protocols that amplify the payoff of absorptive capacity. In contrast, rigid contracts dampen learning and financial returns [47]. These insights suggest that uncertainty not only shapes the efficacy of absorptive capacity but also moderates how family firm protocol mechanisms convert knowledge into superior performance. In this way, the following hypothesis is raised:

H3. Uncertainty in the market has a double moderating effect on the relationship between family firm protocol and the relationship between absorptive capacity and firm performance.

As shown in Fig 1, the conceptual model integrates stewardship-based family firm protocol, absorptive capacity, and market uncertainty.

**Fig 1:**
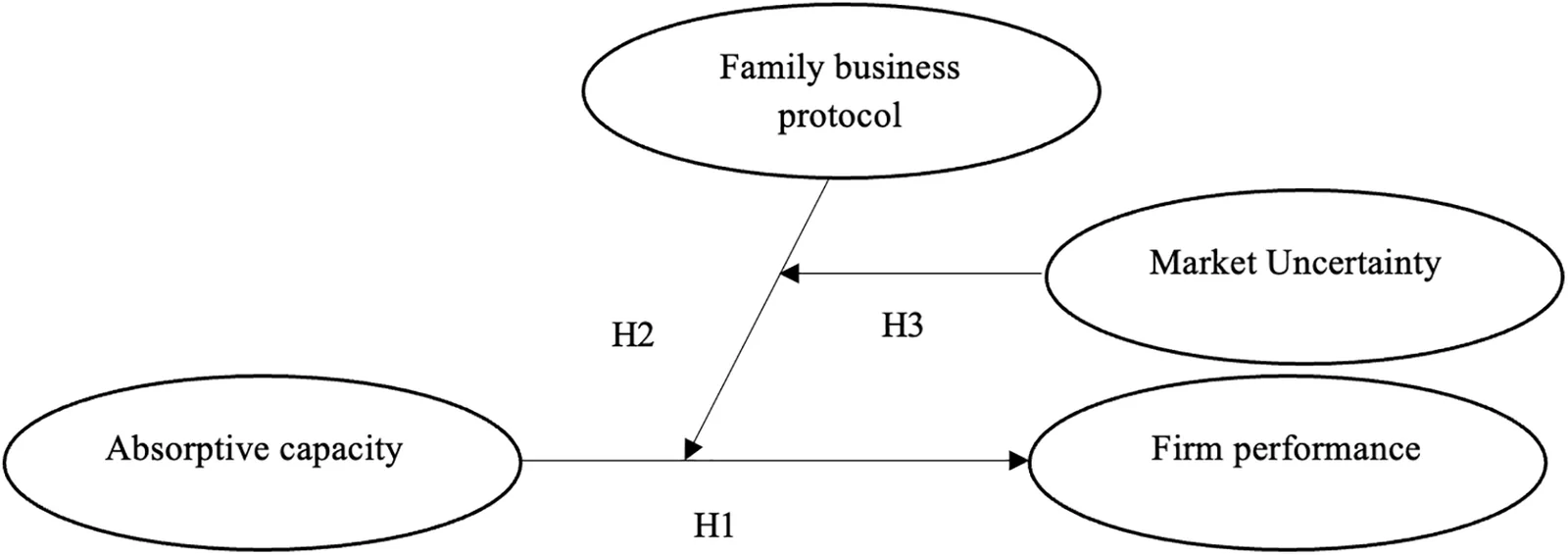
Conceptual model.

## Materials and methods

This study examines how formal family firm protocol structures, particularly the family business protocol, influence the paths of financial performance in born global companies in Colombia, employing a quantitative and longitudinal research methodology. The longitudinal approach enables the investigation of dynamic changes and trends over time, thereby capturing the influence of family firm protocol structures and market uncertainty in highly internationalized settings with limited resources. To precisely record the most recent developments and challenges faced by these businesses, the data collection spans the years 2021 through 2025.

The study was conducted in Bogotá, Cali, Barranquilla, and Medellín, which are Colombia’s main economic cities. Based on official business registrations and industry groups, the companies were identified through a random sampling approach across various cities. Companies had to satisfy the requirements for participation, which included being owned by a family, having internationalized operations within the past five years, and operating across several sectors. The chosen companies were invited to participate annually to compile similar data over five years.

This study was approved by the Research and Ethics Committee of the Faculty of Economic and Administrative Sciences at Pontificia Universidad Javeriana Cali (Approval code: DECA-88-2021) and was conducted in accordance with the principles of the Declaration of Helsinki.

Informed consent was obtained from all participants prior to data collection. Given the low-risk nature of the study and the use of in-person structured interviews with adult participants, consent was obtained verbally. The verbal consent procedure was approved by the Ethics Committee and was documented by the researchers in field records before each interview.

All participants were adults involved in strategic decision-making within their firms; no minors were included in the study. Therefore, parental or guardian consent was not required. The Ethics Committee did not waive the requirement for consent, as informed verbal consent was obtained from all participants.

### Data collection and sampling

There were 400 separate companies in all in the first sample. Data collection was conducted using in person structured questionnaires to ensure direct interaction with respondents. These questionnaires were sent directly to the companies that participated in planned visits. Apart from ensuring high response rates, this approach allowed the researcher to clarify questions anytime they needed. Surveys were distributed annually between January and March. This guaranteed constant application of the data collection process helped manage seasonal fluctuations in business activity.

Participants responded to in-person interviews; most were family members or senior managers involved in strategic decision-making. Every session usually lasted twenty to thirty minutes. Participation was voluntary, and businesses provided informed consent in line with ethical standards for research. Companies were provided with feedback reports that benchmarked their responses against industry averages, fostering involvement and commitment throughout the research process. This was carried out to support ongoing involvement.

The survey consists of four hundred family-owned businesses that are of the kind known as “born global.” Each of these businesses has an age ranging from five to eight years, dating back to their establishment and subsequent entry into international markets. These businesses are active in a variety of economic sectors, with the most significant being the food industry, accounting for approximately 35 percent of the total. Following closely is the automotive industry, which accounts for twenty percent of the population. Then there are other sectors, such as textiles, technology, and services, which together account for forty-five percent of the remaining market. The sectoral distribution enables the analysis of a wide range of organizations that exhibit comparable characteristics in their process of rapid internationalization.

In terms of size, these organizations typically have an average of fifteen employees, which is classified as a small business according to the international classification system. However, upon performing the analysis, it is observed that there is a dispersion of employees ranging from eight to thirty. The socio-economic structure reveals that around seventy percent of the workers in operational positions are female, while the percentage of women working in managerial positions is thirty-five percent. The composition of the direction, on the other hand, is composed of sixty-five percent men. In contrast, the remaining thirty-five percent is comprised of women, indicating a certain gender disparity in the positions that serve as directors. The average age of CEOs is 45 years, with a range that extends from 30 to 60 years. Furthermore, the majority of them, which accounts for sixty percent, have prior experience in the sector. Furthermore, they have an undergraduate level of academic formation, primarily in the areas of engineering, health sciences, and economics, which represents a profile of technical and specialized leadership.

The geographical distribution of these businesses reveals that forty percent are located in Bogotá, thirty percent in Medellín, twenty percent in Cali, and ten percent in Barranquilla. The previous ideas suggest that the majority of business activity is concentrated in the country’s most significant cities. The participation of business owners in educational institutions, commercial sector organizations, and universities is fifty-five percent, and an additional twenty-five percent of business owners participate in programs that provide support and training in areas such as innovation and internationalization.

### Variables

1. Absorptive Capacity: The primary components of absorptive capacity measurement used in this study are the assimilation, transformation, and exploration aspects [48]. The evaluation of the extent to which information and ideas are transmitted across departments within the company is referred to as assimilation. This evaluation reflects the sharing of internal knowledge and expertise. The term “transformation” refers to an organization’s ability to reorganize and connect previously acquired information with new ideas, thereby fostering creative thought. Exploration is the process of evaluating the proactive search for external knowledge, which involves engaging with networks, databases, seminars, and publications to obtain new knowledge relevant to the company’s strategic needs. This three-dimensional scale aligns with contemporary research that emphasizes the multidimensionality of absorptive capacity. It extends beyond simple proxies, such as spending on research and development, to provide a comprehensive understanding of how businesses effectively acquire, internalize, and utilize external knowledge to improve innovation and performance, particularly in dynamic international environments.
2. Firm Performance: The measurement was based on Venkatraman’s [49] firm performance scale, which is a validated and well-established instrument for self-reporting. On a seven-point scale, managers assigned ratings to critical performance dimensions, including profitability and growth. One point signified the lowest relative performance, while seven indicated the highest.
3. Market Uncertainty: This scale, adapted from Homburg et al. [50], is evaluated on a seven-point Likert scale, where one indicates “disagree” and 7 indicates “totally agree.” This scale includes aspects related to volatile prices, dynamics of the market, and changes in technologies.
4. Family Business Protocol: This variable is dichotomic, with 1 indicating the business has a family protocol and 0 indicating the firm does not have a protocol.

### Method

In this research, we implemented a sophisticated analytical methodology known as Dynamic Structural Equation Modeling (DSEM) to evaluate our research hypotheses. Our implementation was consistent with the best practices established by scholars in this field [51]. Some important steps were implemented during the analysis process to guarantee validity and robustness. At first, we exclusively implemented Bayesian estimation techniques, adhering to the rules of Asparouhov et al. (2018) [51], as conventional frequentist maximum likelihood methods frequently demonstrate suboptimal performance when applied to complex structural models as a result of convergence difficulties and slight sample bias. Bayesian estimation provides numerous benefits, including the ability to derive posterior distributions of parameters, which allows for the incorporation of prior knowledge to enhance estimation stability and provides more detailed information about parameter uncertainty.

Lüdtke et al. (2008) [52] were used in our methodology, which included the implementation of latent group-mean centering for each variable. By subtracting the group mean, this procedure isolates within-person fluctuations, allowing us to concentrate on individual change trajectories over time while accounting for personal differences at the group level. This centering increases the interpretability of model parameters, particularly in dynamic contexts where temporal variations are of primary interest.

The Deviance Information Criterion (DIC), a Bayesian analogue of conventional information criteria, was the primary criterion for model evaluation and selection. Model fit and complexity are balanced by DIC, with lower values indicating more parsimonious models with superior predictive accuracy. It is particularly valuable in the context of advanced modeling, as it assists in overcoming the challenges of variance partitioning and model overfitting, which are prevalent in hierarchical and longitudinal data structures. These methodologies are particularly well-suited for the analysis of high-frequency, intensive longitudinal data that is collected through ecological momentary assessments, experience sampling procedures, or ambulatory surveillance (Asparouhov et al., 2017) [53]. In order to assess the accuracy and efficacy of our model, we implemented a variety of statistical indicators:

- Relative Bias: This metric quantifies the deviation of the average estimated parameter from the actual value, with values near zero suggesting minimal bias. A systematic overestimation is indicated by a bias that exceeds 1.
- Standard Error vs. Empirical Standard Deviation (SE/SD): The adequacy of the standard error as an uncertainty measure is evaluated by comparing the model-based standard error to the observed variability of estimates. Comparable SE and SD values indicate reliable estimation of variability.
- Mean Squared Error (MSE): MSE provides an aggregate estimate of the accuracy of parameter estimates by incorporating both bias and variance, with smaller values indicating greater precision.
- Coverage Probability: This evaluates the percentage of confidence intervals that encompass the actual parameter value in multiple samples. Accurate interval estimation is typically indicated by a range of 0.92 to 0.98.
- Statistical Power: The capacity to identify genuine effects is assessed by determining the percentage of parameters with 95% confidence intervals that exclude zero. In the context of regression coefficients across various levels, a higher power indicates a greater sensitivity in identifying meaningful relationships.

## Results

Table 1 displays the descriptive statistics and Pearson correlation coefficients for the principal variables in the research. The findings indicate that firms showed moderate to high levels in the three dimensions of absorptive ability, with exploration reaching a high mean (M = 4.91, SD = 1.14), followed by assimilation (M = 4.83, SD = 1.12) and transformation (M = 4.57, SD = 1.08). Firm performance had a significantly higher mean score (M = 5.02, SD = 1.17), although market uncertainty was assessed as moderate (M = 4.44, SD = 1.21). All measures of absorptive ability showed significant and positive correlations with one another, demonstrating conceptual relationship (r values ranging from.46 to.54, p < .01). Each measure of absorptive capacity shown a substantial positive link with business performance: assimilation (r = .43), transformation (r = .39), and exploration (r = .37), all with p < .01.

**Table 1:** Correlation matrix.

Variable	Mean	SD	1	2	3	4	5
1. Assimilation	4.83	1.12	1				
2. Transformation	4.57	1.08	.54**	1			
3. Exploration	4.91	1.14	.46**	.52**	1		
4. Firm Performance	5.02	1.17	.43**	.39**	.37**	1	
5. Market Uncertainty	4.44	1.21	.30**	.28**	.33**	.25**	1

### Model estimation and fit

To evaluate the model’s adequacy, fit statistics to Bayesian estimation and DSEM were analyzed (See Table 2). The Deviance Information Criterion (DIC) was the primary measure of overall model fit, with a score of 11,237.54. Lower DIC values signify superior model parsimony and prediction accuracy, indicating an appropriate model fit. The ratio of standard error to standard deviation (SE/SD) was about 1.02, indicating that the model’s uncertainty estimation aligns with the observed variability, which indicates reliable parameter estimation. The relative bias was under ±0.05, indicating minimal estimation bias and corroborating the precision of the Bayesian estimations.

**Table 2:** Model fit statistics.

Fit Statistic	Value	Interpretation
Deviance Information Criterion (DIC)	11237.54	Acceptable fit (lower indicates better fit)
SE/SD Ratio	~1.02	Indicates reliable uncertainty estimation
Relative Bias	<±0.05	Minimal estimation bias
Mean Squared Error (MSE)	Low	Suggests high estimation accuracy
Coverage Probability	0.94	Indicates accurate interval estimation
Statistical Power	0.88–0.94	Sufficient for detecting true effects

Additionally, the mean squared error (MSE) was minimal, signifying higher estimation accuracy through the equilibrium of variance and bias in the parameter estimations. The coverage probability, defined as the proportion of 95% credible ranges including the correct parameter values, was 0.94, which is within the advised range of 0.92 to 0.98, validating the model’s interval estimations as well as the adjusted. Finally, the statistical power varied from 0.88 to 0.94, considered enough for identifying genuine effects and reinforcing the robustness of the inferential results (Asparouhov et al., 2018; McNeish & Hamaker, 2020).

Hypothesis 1 affirms that the three dimensions of absorptive capacity, such as assimilation, transformation, and exploration, positively influence firm performance. As shown in Table 3, all three dimensions were significant predictors of firm performance. Therefore, the results support the H1, confirming that higher levels of absorptive capacity are associated with improved firm performance.

**Table 3:** Main effects of absorptive capacity on firm performance (H1).

Predictor	Estimate (β)	SE	95% Credible Interval	p (β ≠ 0)
Assimilation	0.32	0.08	[0.16, 0.47]	> 0.95
Transformation	0.28	0.07	[0.14, 0.41]	> 0.95
Exploration	0.24	0.09	[0.08, 0.41]	> 0.95

Hypothesis 2 affirms that the existence of a family business protocol moderates the relationship between absorptive capacity and firm performance. As indicated in Table 4, interaction terms for assimilation and transformation with family protocol were significant, whereas the interaction with exploration was marginally non-significant.

**Table 4:** Moderating effects of family business protocol (H2).

Interaction Term	Estimate (β)	SE	95% Credible Interval	p (β ≠ 0)
Assimilation × Family Protocol	0.21	0.07	[0.08, 0.34]	> 0.95
Transformation × Family Protocol	0.18	0.08	[0.03, 0.33]	> 0.90
Exploration × Family Protocol	0.09	0.06	[-0.02, 0.21]	~0.85

These findings partially support H2, indicating that the presence of a family protocol strengthens the impact of specific dimensions of absorptive capacity, especially assimilation and transformation, on firm performance.

Hypothesis 3 proposes a double moderation, suggesting that market uncertainty moderates the interaction between absorptive capacity and family firm protocol in predicting firm performance. As shown in Table 5, two of the three-way interaction terms were significant (assimilation and transformation), while the effect for exploration was not statistically significant.

**Table 5:** Double moderation effects of market uncertainty (H3).

Three-way Interaction Term	Estimate (β)	SE	95% Credible Interval	p (β ≠ 0)
Assimilation × Family Protocol × Market Uncertainty	0.15	0.06	[0.04, 0.27]	> 0.95
Transformation × Family Protocol × Market Uncertainty	0.10	0.05	[0.01, 0.20]	> 0.90
Exploration × Family Protocol × Market Uncertainty	0.05	0.04	[-0.03, 0.13]	~0.75

These results provide partial support for H3, highlighting that market uncertainty further strengthens the moderating effect of family protocol—primarily for assimilation and transformation dimensions.

Finally, Table 6 provides a summary of the results obtained from the hypothesis testing. Hypothesis 1 received complete support, demonstrating that all three aspects of absorptive capacity, such as assimilation, transformation, and exploration, positively affect business performance. Hypothesis 2 obtained some support, because the moderating influence of the family business protocol was significant for assimilation and transformation, but not for exploration. Hypothesis 3 received limited support: market uncertainty enhanced the moderating effect of the family protocol on specific measures of absorptive ability, but not globally.

**Table 6:** Summary of hypothesis testing.

Hypothesis	Description	Supported
H1	Absorptive capacity (assimilation, transformation, exploration) → Performance	Yes
H2	Family protocol moderates the Absorptive capacity (ACAP) → Performance relationship	Partial
H3	Market uncertainty moderates the moderation (double moderation)	Partial

## Discussion

This study looked at how absorptive capacity affects firm performance in born worldwide family businesses and how market volatility and family business practices affect this connection. The results offer detailed information on the many functions of the absorptive capacity dimensions and the governing mechanisms that influence their efficacy through the use of dynamic structural equation modeling.

First, since all three dimensions assimilation, transformation, and exploration—have a major beneficial impact, the results validate that absorptive ability is a crucial factor in determining business performance. This result supports the idea that knowledge-based skills are essential to maintaining competitive advantages in family businesses with a global focus. According to earlier studies, the capacity to absorb and modify outside knowledge seems to be especially important for converting information into workable plans and operational enhancements. The beneficial effect of exploration further implies that, even in companies with strong internal linkages and route dependency, ongoing contact with new knowledge sources is still crucial.

Second, the family business protocol’s moderating function shows how governance structures influence how information is used to achieve performance goals. The findings indicate that the impacts of assimilation and transformation—but not exploration—are considerably strengthened by family procedures. This disparity implies that structured family agreements could be more successful in promoting internal information processing routines than in encouraging exploratory behaviors, which frequently call for greater risk tolerance and receptivity to outside influences. According to stewardship theory, family protocols may improve coordination, trust, and goal alignment, which makes it easier for information to be integrated and used throughout the company. However, the reduced moderating impact may be explained by the same processes that limit experimentation and boundary-spanning behaviors related to exploration.

Third, the contextual relevance of market uncertainty is shown by the twofold moderation hypothesis’ partial support. The relationship between absorptive capacity and family procedure is more noticeable in situations of increased uncertainty, especially when it comes to absorption and transformation. According to this research, well defined family governance structures serve as stabilizing mechanisms that help businesses better process and use information in unstable external settings. Family protocols seem to offer a framework for disciplined adaptation, enabling businesses to react cogently to environmental shocks rather than increasing risk aversion. However, the lack of a meaningful three-way interaction for exploration suggests that uncertainty does not consistently improve all learning-related processes, supporting the idea that exploratory activities are still susceptible to environmental complexity and governance limitations.

When considered collectively, these results highlight the significance of differentiating between absorptive capacity aspects and acknowledging governance systems as situational facilitators rather than consistent performance amplifiers. The study provides a more detailed explanation of performance variation among born global family enterprises and extends our knowledge of how family business protocols interact with organizational learning processes and external variables.

## Conclusion

This study looked at the link between absorptive capacity and firm performance in multinational corporations that were founded by families, as well as how market uncertainty and family business customs have shaped this relationship over time. The results show that, although its efficacy varies across dimensions and contextual conditions, absorptive capacity is an important performance-related capability in internationally oriented family businesses, according to a longitudinal design and dynamic structural equation modeling.

The findings support the idea that knowledge-based capabilities lead to better success in early internationalizing family firms by demonstrating a positive correlation between firm performance and all three aspects of absorptive capacity: assimilation, transformation, and exploration. These results demonstrate that, even in highly internationalized and resource-constrained environments, both internal information processing systems and external knowledge-seeking activities are important for financial performance.

The analysis shows that the existence of a formal family business procedure has a dependent moderating influence in addition to these direct effects. In particular, there is no statistically significant moderation for exploration, although family procedures enhance the performance benefits of assimilation and transformation. This trend indicates that while more exploratory and boundary-spanning learning activities seem to be less receptive to such formal frameworks, structured family governance systems are especially successful in strengthening internal knowledge integration and recombination processes.

The data also shows that the moderating function of family protocols is conditioned by market uncertainty. The favorable relationship between family protocols and the absorption and transformation aspects of absorptive ability is particularly noticeable at increasing levels of uncertainty. This result emphasizes the situational usefulness of formal family governance structures in unstable contexts, where formalized frameworks for decision-making may facilitate more efficient use of internal information. The lack of a meaningful three-way interaction for investigation, however, highlights the limitations of formalization in situations that call for adaptability and experimentation.

Finally, our findings add to the literature on family business and foreign entrepreneurship by indicating that family governance structures are neither consistently restrictive nor generally advantageous. Their performance consequences vary depending on the precise learning processes involved and the amount of environmental uncertainty encountered by the company. By combining absorptive capacity dimensions, family business protocols, and market uncertainty into a single longitudinal framework, this study provides a more nuanced understanding of how governance structures influence performance-related learning processes in family-owned global enterprises.

## Supporting information

S1 File Data. (XLSX)
